# Biochar Particles Obtained from Agricultural Carob Waste as a Suitable Filler for Sustainable Biocomposite Formulations

**DOI:** 10.3390/polym14153075

**Published:** 2022-07-29

**Authors:** Giulia Infurna, Luigi Botta, Marco Maniscalco, Elisabetta Morici, Giuseppe Caputo, Salvatore Marullo, Francesca D’Anna, Nadka Tz. Dintcheva

**Affiliations:** 1 Dipartimento di Ingegneria, Università degli Studi di Palermo, Viale delle Scienze, Ed. 6, 90128 Palermo, Italy; giulia.infurna@unipa.it (G.I.); marco.maniscalco02@unipa.it (M.M.); elisabetta.morici@unipa.it (E.M.); giuseppe.caputo01@unipa.it (G.C.); 2 ATeN Center, Università di Palermo, Viale delle Scienze, Ed. 18, 90128 Palermo, Italy; 3 Dipartimento STEBICEF, Sez. Chimica, Università degli Studi di Palermo, Viale delle Scienze, Ed. 17, 90128 Palermo, Italy; salvatore.marullo@unipa.it (S.M.); francesca.danna@unipa.it (F.D.)

**Keywords:** biocomposites, biochar particles, circular economy, radical scavenging activity, photo-oxidation resistance

## Abstract

In the context of sustainable and circular economy, the recovery of biowaste for sustainable biocomposites formulation is a challenging issue. The aim of this work is to give a new life to agricultural carob waste after glucose extraction carried out by a local factory for carob candy production. A pyrolysis process was carried out on bio-waste to produce biofuel and, later, the solid residual fraction of pyrolysis process was used as interesting filler for biocomposites production. In this work, biochar particles (BC) as a pyrolysis product, after fuels recovery of organic biowaste, specifically, pyrolyzed carobs after glucose extraction, were added on poly(butylene-adipate-co-terephthalate), (PBAT), at two different concentrations, i.e., 10 and 20 wt%. The BC have been produced using three pyrolysis processing temperatures (i.e., 280, 340 and 400 °C) to optimize the compositions of produced solid fractions and biofuels. The resulting particles from the pyrolysis process (BC280, BC340 and BC400) were considered as suitable fillers for PBAT. Firstly, the BC particles properties were characterized by elemental composition and spectroscopy analysis, particle size measurements and evaluation of radical scavenging activity and efficiency. Moreover, PBAT/BC composites were subjected to analysis of their rheological and thermal behavior, morphologies and mechanical properties. In addition, accelerated weathering, monitored by both tensile test and spectroscopic analysis, was carried out, and obtained results show that the biochar particles can exert a beneficial effect on photo-oxidation delay of PBAT matrix.

## 1. Introduction

The valorization of organic waste and the development of bio-based materials, containing naturally occurring constituents, are the trends undertaken by several current research programs and initiatives as key action for sustainable development [1,2,3]. Moreover, the effort towards the production of new sustainable biomaterials is justified by the increasing demand for environmentally sustainable products [4,5,6,7,8,9]. Therefore, the formulation of bio-based materials, having good properties and performance in service and controlled end-of-life, is imperative for an effective circular economy [7,10,11,12]. In the contest of circular economy, pyrolysis, that is, a thermal decomposition process in inert atmosphere, could be a challenging process to convert organic waste into three different new resources: synthetic gas (a mixed gas phase of hydrocarbons), oil (mix of heaviest hydrocarbons) and biochar, a solid waste. Depending on thermal degradation conditions of pyrolysis, such as temperature range, pressure and residence time, the proportion of these three products changes [13,14,15]. Fast pyrolysis (high pressure and high temperature) is the most efficient method to produce biofuel [16,17,18], however slow pyrolysis tends to yield higher proportion of solid phase, besides being energetically cost-effective, and for this reason is the most used to recover waste [16,19,20].

Biochar, the solid fraction, preferential product of slow pyrolysis, is a carbon rich material, usually employed as soil recover to enhance its fertility [13,14,21]. Nowadays BC has attracted increasing interest as filler for polymer [22,23,24,25,26] and bio-polymer composites [27,28,29] due to several factors. In fact, biochar is characterized by a high amount of aromatic C and condensed aromatic structure, in different forms, including graphite; it also contains hydrogen, oxygen, nitrogen Sulphur calcium and silicon and the proportion between them depends on two main factors: the nature of biomass employed as well as the temperature used during pyrolysis. Sizirici et al. [14] show how the proportion between carbon and calcium and the other components increase as the pyrolysis temperature increases. Biochar structure can be tuned depending on the pyrolysis condition; in fact, varying temperature and oxygen flow, the internal porosity and the presence of leftover functional groups can be tailored, working to the surface area [13,20]. Moreover, biochar can increase mechanical and thermal stability and can provide great electric properties as well as barrier properties added to polymer matrix [25,28,30,31]. Suiyi Li et al. [32] show how dispersing nano sized biochar particles in UHMWPE results in the particles forming a segregated conductive network, reducing the percolation threshold of the composites. Carbonaceous particles, such graphene, carbon black and carbon nanotubes, have gained great interest also due to their protective ability through different mechanisms: first the ability to absorb UV radiation due to their dark color and second due to their radical scavenging activity [33,34]. As is well known, the carbonaceous particles can absorb UV photo-radiation and dissipate this energy without matrix damage. Interestingly, radical scavenging activity of this particles can be explained considering that a large amount of carbon atoms presented in these particles have *sp^2^* hybridization and some of them are able to change their hybridization to *sp^3^* upon irradiation, achieving scavenger ability. Botta et al. [35] showed that biochar particles are also able to scavenger radical, retarding photo-oxidation phenomena in bio-polyester composites.

Poly(butylene adipate-co-terephthalate), PBAT, is a random biodegradable thermoplastic bio-polyester obtained by polycondensation of butanendiol (BDO), adipic acid (AA) and terephtalic acid (PTA). PBAT show a good biodegradability due to the aliphatic unit in the molecule chain and excellent mechanical properties due to the presence of aromatic units; PBAT has mechanical properties comparable with low density polyethylene, LDPE [36,37]. Mechanical properties of PBAT could be affected by the adipic acid and therpethalic monomer ratio; in fact, Young Modulus increases, and elongation break decreases with the increase of terephthalate units [38,39]. A good biodegradability, the mechanical properties comparable with LDPE, makes PBAT a good candidate for filmability both to packaging application and to mulch film production [40].

Considering circular economy principles, in this work biochar particles obtained from agricultural carob waste have been considered as a suitable filler for sustainable biocomposites formulations. Particularly, biochar particles, obtained as solid residue of a slow pyrolysis process of carob waste, were used as filler for the preparation by melt mixing of PBAT based biocomposites. Specifically, three different biochar particles, as result of three different temperatures of pyrolysis, were incorporated in the biodegradable polymeric matrix at two different concentrations, i.e., 10 and 20% wt/wt. To fully investigate biochar particles properties, preliminary characterization of particles dimension, ATR spectra, radical scavenging activity and elemental analysis were conducted. Indeed, to characterize the biocomposites the morphological, thermal, mechanical and rheological characterizations were carried out. Finally, the PBAT composites were exposed to accelerate weathering equipment and monitored by means of spectroscopic analysis as a function of irradiation time to evaluate the photo-oxidation resistance of the produced biocomposites. All performed characterizations aim to investigate the relationship between morphological structure of BC particles, particle/matrix interaction between biocomposites properties, taking into account the circular economy principles.

## 2. Materials and Methods

### 2.1. Materials

Poly(butylene adipate-co-terephthalate), (PBAT; commercial Ecoflex^®^ F Blend C1200, BASF, SE, Ludwigshafen, Germany) is a film grade with a melt flow rate (MFR) of 2.7–4.9 g/10 min (190 °C, 2.16 kg), a density in the range of 1.25–1.27 g/cm^3^ and a melting temperature in the range of 110–120 °C.

**Scheme 1:**
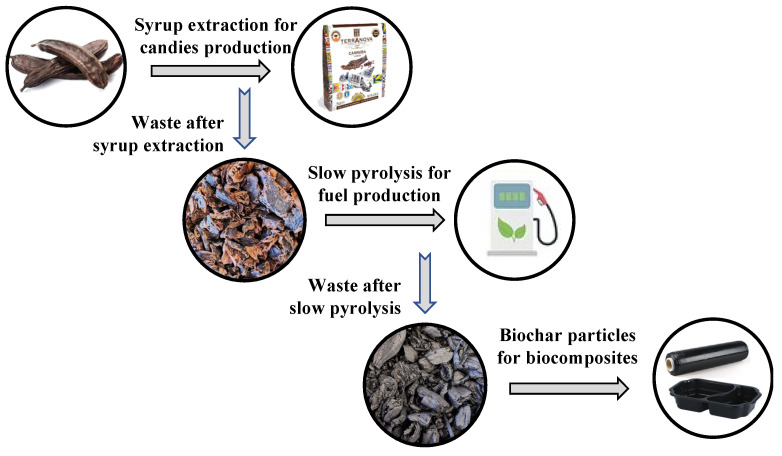
Flow, followed in this work, to produce biochar particles and sustainable biocomposites.

Biochar particles (BC) have been produced using carob waste after syrup extraction, for carob candy production, and slow pyrolysis, for fuel production, as shown in Scheme 1. The BCP has been produced by slow pyrolysis, as a second level waste after pyrolysis process carried out at three different temperatures, i.e., 280, 340 and 400 °C, as reported in our previous work [41]. In this work the biochar particles as a result of pyrolysis conducted at 280, 340 and 400 °C are named BC280, BC340 and BC400, respectively. The residual biochar from these three different pyrolysis conditions was milled at the same process condition.

### 2.2. Biocomposite Formulation

**Scheme 2:**
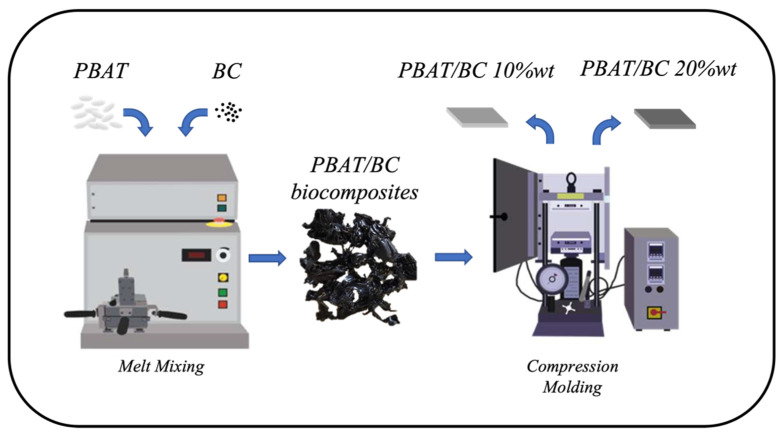
Scheme of Bio-composite production.

The bio-composite formulation was carried out by means of a batch mixer (Brabender, Model PLE330, Duisburg, Germany)at 170 °C, with a mixing speed of 50 rpm for 5 min. Before compounding, PBAT and BC were dried at 60 °C under vacuum to avoid hydrolysis during compounding. The three different samples BC280, BC340 and BC400 were added at 10 wt% and 20 wt%. Then the compound was pelletized. In addition, the neat PBAT was subjected to the same processing condition in order to be comparable with the composites. After 24 h of drying in an under vacuum oven at 60 °C, to avoid hydrolysis process, thin films (thickness of about 200 μm) of neat PBAT and of all composites were obtained through compression molding by means of Carver Laboratory Press (Carver, Wabash, IN, USA) at a pressure of 1500 psi for 5 min at 170 °C. In Scheme 2 the production process of bio-composites is represented.

### 2.3. Characterization of BC

The size of the three different biochar particles were measured using a Malvern Mastersizer 2000 granulometer with an ultrasound treatment. The Mastersizer 2000 granulometer was equipped with Malvern Hydro 2000 MU that uses a stirrer for the dispersion of 1 gr of samples into 800 mL of deionized water. All the analysis were carried out at stirrer velocity of 2000 rpm, after 5 min of sonication. Diameter size distribution was plotted after performing measurements onto six different samples. From this measurement, the particle distribution curves were extrapolated. Three factors, d10, d50 and d90, were calculated, which represent the maximum diameter value of 10%, 50% and 90% of the particles, respectively.

To understand the chemical composition, ATR-FTIR analysis was carried to the BC (Perkin-Elmer FT-IR/NIR Spectrum 400, Waltham, MA, USA), and 8 acquisitions were performed for each measurement.

The elemental analysis, i.e., the determination of carbon, hydrogen and nitrogen has been performed by means of a TruSpec CHN LECO CHN 628 (ASTM D5373) analyzer. In this case, about 100 ÷ 150 mg of dried, grinded and homogenized sample was introduced in the apparatus.

The 1,1-diphenyl-2-pycryl (DPPH, supplied by Sigma Aldric) free radical scavenging assay was carried out [33,42,43]. First, a methanol solution of DPPH (10^−4^ M) was prepared, then 1 mg of solid was placed in 2 mL of this solution for 24 h at 25 °C. The solutions were kept at 25 °C during the time required by the measurement. Then the supernatant liquid was removed, and the UV-vis spectrum was recorded at different step times, achieving 24 h in a Beckmann DU-800 spectrometer. Spectra were recorded on a spectrophotometer equipped with a Peltier temperature controller. Moreover, a study of BC concentration was performed, adding 1, 2, 5 and 10 mg of solid in the methanol solution of DPPH (10^−4^ M), recording spectra at the end of 24 h. Scavenging activities were determined from the drop in absorbance at 517 nm of each sample compared with that of the DPPH solution in the absence of contact with the material. Scavenging efficiency values were calculated by Equation (1):

(1) $$\mathrm{Radical}\;\mathrm{Scavenging}\;\mathrm{Efficiency}\;(\%)=\frac{A-B}{A-0.1}\times 100$$

where *A* is the absorbance at 517 nm of the DPPH solution and *B* is the one of the DPPH solution after contact with the solid.

### 2.4. Characterization of Biocomposites

Rheological tests were performed using a strain-controlled rheometer (mod. ARES G2 by TA Instrument, New Castle, DE, USA) in parallel plate geometry (plate diameter 25 mm). The complex viscosity (η*) and storage (G′) and loss (G″) moduli were measured by performing frequency scans from ω = 10^−2^ to 10^2^ rad/s at same processing temperatures. The strain amplitude was γ = 5%, which preliminary strain sweep experiments proved to be low enough to be in the linear viscoelastic regime.

The microstructure of the bio-composites was investigated using a Scanning Electron Microscope (SEM, Quanta 200 ESEM, FEI, Hillsboro, OR, USA). Prior to SEM analysis, samples were fractured in liquid nitrogen. The fractured surface of each sample was sputtered (Scancoat Six Edwards, Crawley, UK), with a thin layer of gold under argon atmosphere for 90 s, in order to avoid electrostatic charging under the electron beam.

Enthalpy of fusion of all samples was measured by DSC by means of Shimadzu (Japan) DSC-60 apparatus, with a heating rate of 10 °C/min from T ambient to 170 °C, as the average of five measurements. All samples of similar weight (~7 mg) were subjected to heating/cooling/heating and the thermal parameters were evaluated on the second heating scan, erasing the previous thermal history. The crystallinity grade of PBAT, *χ_c_*, was calculated using the following equation:

(2) $$\chi_{c}=\frac{\Delta H_{m}}{{\Delta H}_{100}\times W_{p}}\times 100$$

where $\Delta H_{m}$ is the enthalpy of fusion, ${\Delta H}_{100}$ is the enthalpy of fusion of 100% crystalline polymer and $W_{p}$ is the weight fraction of polymer. For PBAT ${\Delta H}_{100}$ = 114 J g^−1^ [44].

Tensile tests were carried out using a Universal Testing Machine (Instron model 3365, Rochdale, UK), following ASTM D882 method, on rectangular samples. The tests were performed, using tensile speed at 1 mm/min for 1 min in order to evaluate the Young’s Modulus and then the velocity was increased to 10 mm/min until sample breakage. The average values for elongation at break, EB, for elastic modulus, E, and for tensile strength, TS, were calculated.

Dynamic mechanical thermal tests (DMTA) were carried out in tensile configuration by means of a dynamic mechanical analyzer model DMA +50 (Metravib, Limonest, France). The test was performed for three samples (10 mm 30 mm) of each composite from room temperature to 120 °C, at a heating rate equal to 2 °C/min. For some composite measurement was stopped before 120 °C. The frequency was set at 1 Hz, and a previous static displacement of 2 × 10^−5^ m and dynamic displacement were set equal 1 × 10^−5^ m, respectively.

Statistical analyses of the data were performed through one-way analysis of variance, and when applicable, data were compared using the Student’s *t*-test. *p*-value < 0.05 was considered statistically significant.

All samples were subjected to a photo-oxidation process by means of a Q-UV/se accelerated weathering tester (Q-Labs Corp., Westlake, OH, USA) containing eight UVB-313 lamps. The samples were exposed at 70 °C to an irradiance of 0.89 W/m^2^ (at a wavelength λ = 313 nm) and monitored every 24 h.

To understand the chemical composition and to follow the change in functional groups as during the accelerating weathering, FTIR-ATR analysis was carried out both on neat PBAT matrix and on the PBAT/BC composites (Perkin-Elmer FT-IR/NIR Spectrum 400, Waltham, MA, USA). Moreover, tensile test of photo-oxidated samples was performed, to monitor the variation of main mechanical properties.

## 3. Results and Discussion

### 3.1. BC Properties

**Figure 1:**
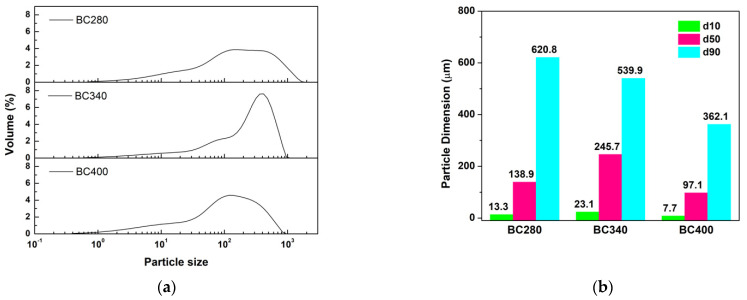
(**a**) Distribution of size dimensions and (**b**) fraction of particles for BC produced at different temperatures, i.e., 280, 340 and 400 °C.

The size of BC particles, obtained at the three different pyrolysis temperature, were measured using a granulometer with an ultrasound treatment, and thanks to this analysis, it was possible to obtain the size distribution curves of the particles, see Figure 1a. The difference in dimensions of the three different BC is a direct result of pyrolysis conditions and the consequent fragility, since the milling conditions were the same for the three particles. The factors d10, d50 and d90, that represent the maximum diameter values of 10%, 50% and 90% of the particles, respectively, were also calculated, see Figure 1b. It is possible to suppose, looking at the decreasing of d90, as the increase of the pyrolysis temperature, and at the size distribution curves, that globally BC400 shows the minimum dimension with respect to the other particles. For d50, the maximum value was obtained for the particles pyrolyzed at 340 °C. Interestingly, the d10 maintains almost the same value for the three different particles.

Furthermore, CHN elemental analysis of the carob waste flour and BC particles, pyrolyzed at different temperatures, was performed, and in Table 1 obtained results are reported. The results highlight that, according to the literature [14], the carbon content in BC increases as the pyrolysis temperature increases, due to the carbonization and thermochemical decomposition of the biomass. Thus, cellulose, hemicellulos and lignin structures involve a different resistant graphitic bond, which can be related to different particles’ dimension after the same milling process.

**Figure 2:**
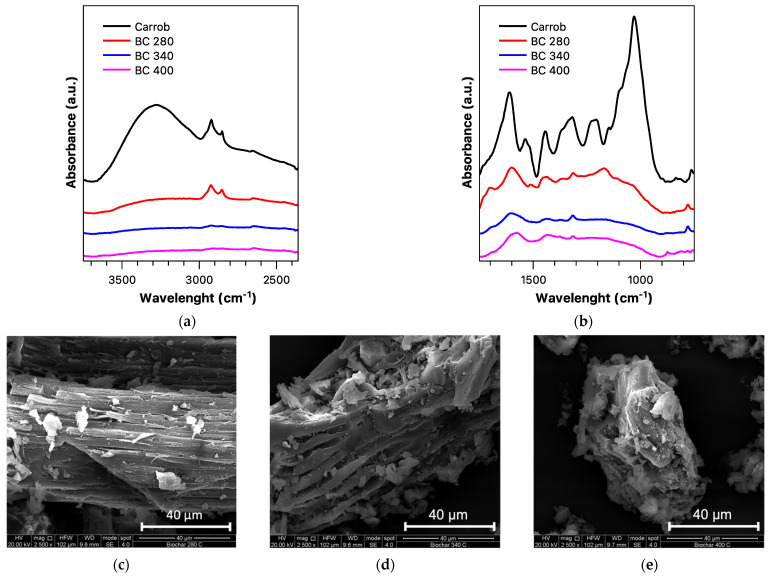
(**a**) 3750–2360 cm^−1^ (**b**) 1750–750 cm^−1^ ATR-FTIR spectra (a.u.) of carob waste after syrup extraction and produced biochar particles after slow pyrolysis at different temperatures, and SEM images of BCP obtained pyrolyzing carob waste at (**c**) 280 (**d**) 340 and (**e**) 400 °C.

Figure 2a,b shows the FT-IR spectra of carob feedstock and BC as the pyrolysis temperature increases. The broad band at 3600–3000 cm^−1^, related to -OH vibration, and the peaks at 2915 and 2860 cm^−1^, related to saturated symmetrical and asymmetrical -CH stretching, significantly reduce from feedstock to biochar structure and even more with the increase of pyrolysis process temperature. The band at 1610–1620 cm^−1^ represents the C=C stretching vibration, related to the presence of aromatic structures. Several other signals, i.e., saturated δ_C-H_ at around 1370–1440 cm^−1^, prove the presence of polysaccharides, aromatic and organic molecules that could be related to ligno-cellulosic structure. All these signals visible in ATR-FTIR of feedstock significantly disappear with the increase of pyrolysis process temperature, and the functional groups decrease with the increase of the pyrolysis temperature. Thus, the carob feedstock was dehydrated upon increases in pyrolysis temperature. Therefore, at 400 °C the organic groups are almost removed and no bands, but only shoulders, are noticed in ATR-FTIR spectra shown in Figure 2a,b. This result is in accordance with SEM micrographs shown in Figure 2c,d, in which the lignocellulosic structure that is still conserved for BC280 and BC340 is completely lost for BC400, which appears more as a carbonaceous particle.

**Figure 3:**
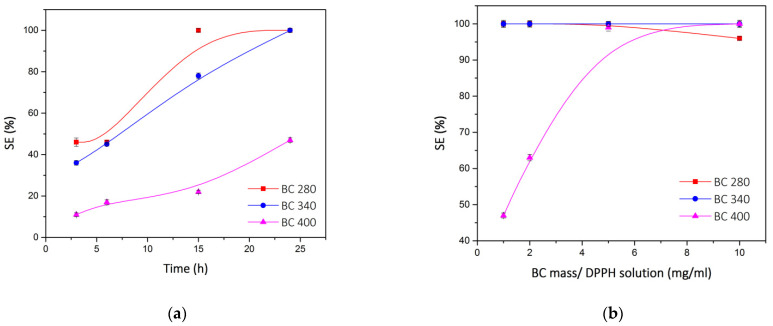
Scavenging efficiency of (**a**) 1 mg/mL of solid as function of time (left) and (**b**) varying the content of solid in DPPH solution after 24 h (right).

To assess the chemical activity of BC particles, their radical scavenging activities were investigated. Specifically, the radical scavenging activities of BC were determined from the drop in absorbance at 517 nm of each sample due to the interaction between DPPH and BC, when dispersed in methanol solution. First, the kinetics of scavenging activity was studied, monitoring scavenging efficiency as a function of time, see Figure 3a, taking as constant the amount of BC. The plot reported in Figure 3a shows similar scavenging efficiency going from BC280 to BC320, although in the latter case, a slightly slower scavenging kinetic was observed. Conversely, for BC400 a marked decrease in both scavenging efficiency and rate occurs, with scavenging efficiency never exceeding 40% even after 24 h. The above result perfectly agrees with information gained from FT-IT and SEM investigation, accounting for a carbonaceous nature of the BC400 sample. Then, an analysis was conducted increasing the BC amount and performing measurement after 24 h. Thanks to the presence of some residual functional groups in BC280 and BC340, particles showed faster kinetics in scavenging free radicals in the solution, and the scavenging efficiency almost remained constant with the increase of BC amount, see Figure 3b. Instead, to achieve approximatively 100% of scavenging efficiency for BC400 it is necessary to raise the amount to 5 mg of BC400 in 2 mL of DPPH solution.

### 3.2. Biocomposites Properties

**Figure 4:**
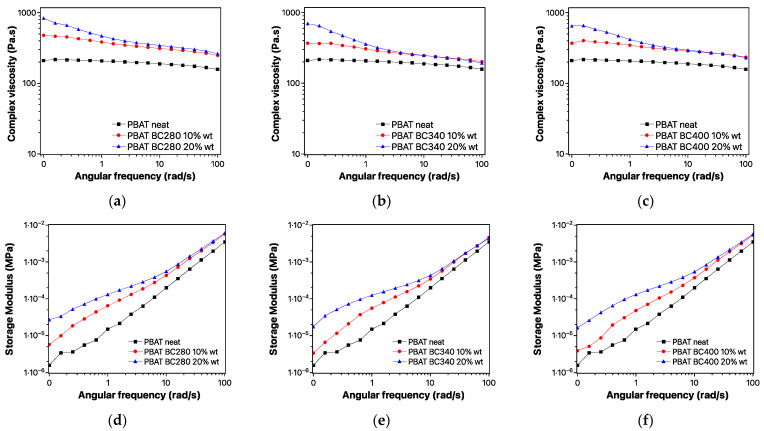
Rheological behavior of bio-composites: complex viscosity of (**a**) PBAT BC280, (**b**) PBAT BC340 and (**c**) PBAT BC400 and storage modulus of (**d**) PBAT BC280, (**e**) PBAT BC340 and (**f**) PBAT BC400 composites.

The rheological behavior of pristine PBAT and of all the PBAT biocomposites was evaluated through oscillatory measurements. In Figure 4a–c, the trends of complex viscosity for each system as a function of frequency are reported. A progressive increase of the complex viscosity of the bio-composites with the increase of the amount of incorporated filler can be observed. It is worth noting that a yield stress behavior can be noticed at low frequency for all composites, and this phenomenon is more evident for the composites at the highest content of BC. The increase of complex viscosity with BC content also reflects an increase in storage modulus G’, see Figure 4d,e. Again, the effect of the BC content inclusion was higher as the filler content in the polymer matrix increases, and is more pronounced at low frequencies. In fact, at low frequencies, an increase of one order of magnitude of storage modulus is detected when 20 wt% of filler is added to the PBAT matrix. This behavior is generally related to a limitation of macromolecules relaxation, and thus due to a high polymer–filler and filler–filler interactions. Therefore, with the increase of BC-content, a reduction of chain mobility of PBAT probably occurs. The Newtonian behavior, exhibited by pure PBAT, tends to progressively disappear as the BC amount in the composites increases, for all pyrolysis temperatures.

**Figure 5:**
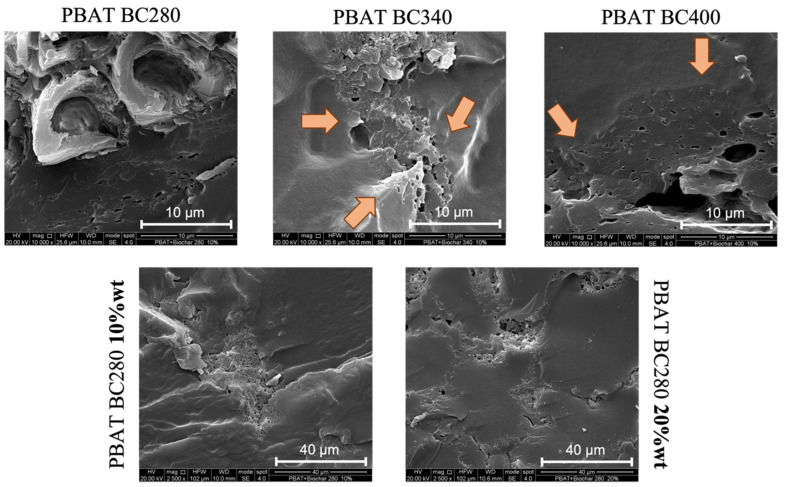
SEM micrographs of biocomposites varying pyrolysis temperature of biochar (top) and varying biochar concentration (down).

Figure 5 shows the micrographs of the nitrogen-fractured surface of PBAT/BC composites comparing composites formulated with particles obtained at the three different pyrolysis temperatures. Moreover, for sample PBAT/BC280 the micrographs varying the content of particles in the biocomposites are shown. The morphology of the fractured surface of PBAT/BC composites suggest in all system a good matrix/filled interfacial adhesion and this can likely be ascribed to the affinity between PBAT matrix and the filler. As noticeable, the affinity does not depend on BC content and on the pyrolysis temperature at which the composites were obtained. Additionally, in this case, as already observed and discussed before for BC particles, the filler showed a pronounced lignocellulosic structure at lower pyrolysis temperature, in contrast to the one obtained at 400 °C that appears predominantly carbonaceous.

**Figure 6:**
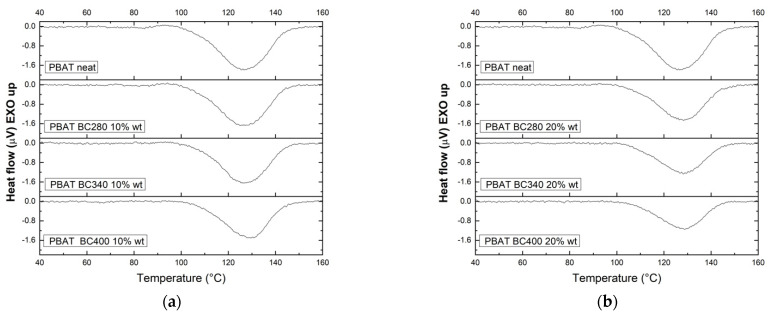
DSC curves of second heating scan of PBAT/BC biocomposites containing BC at (**a**) 10 wt%. and (**b**) 20 wt%.

Figure 6 shows the DSC thermograms recorded with a heating rate of 10°/min for all investigated samples, and in Table 2, the main properties resulting from thermal cycle are listed. The presence of BC particles causes a slight increase of T_m_ with respect to neat PBAT, and for the composites with 10 wt% of loading, the T_m_ slight increase with the increasing of pyrolysis temperature. Furthermore, the T_m_ results are almost constant for the composites with 20 wt% of BC. However, the total content of crystalline phase in bio-composites is significantly reduced by adding BC, and it reduces with the increase of particles loading, and with the increase of pyrolysis temperature at which the biochar particles are produced. Thus, the presence of disperse fillers does not work as a nucleating agent for PBAT matrix, and, in this case, it hampers the crystallization process.

**Figure 7:**
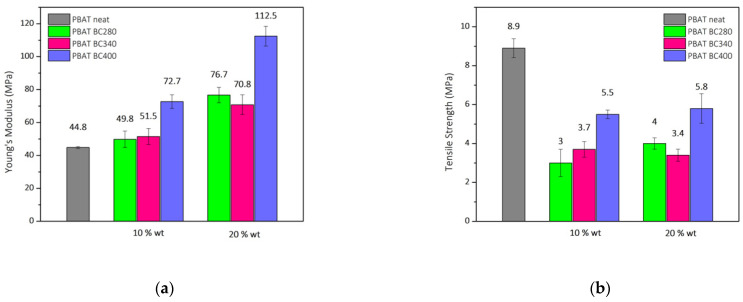

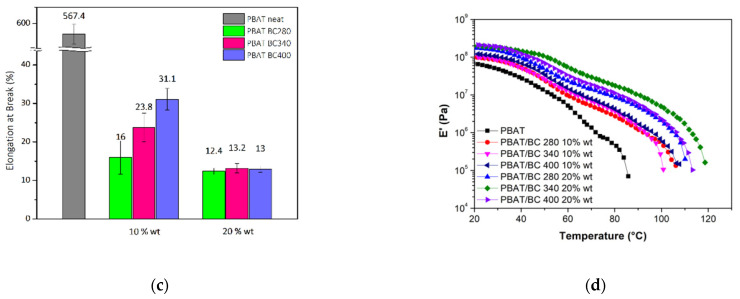
Main mechanical properties, i.e., (**a**) Young’s Modulus, E, (**b**) tensile strength, TS, and (**c**) elongation at break, EB, and (**d**) storge modulus, E′, vs. temperature of PBAT/BCP biocomposites.

The mechanical properties of PBAT-based composites were evaluated through tensile test and dynamic-mechanical analysis; the obtained mechanical properties, Young’s Modulus, tensile strength, and elongation at break are reported in Figure 7a–c. The trends of E’ vs. T for all composites is shown in Figure 7d. Tensile values, i.e., E and TS, increase as the BC load increases and as the pyrolysis temperature increases. As was expected, the ductile behavior of PBAT turns out to be a rigid behavior adding carbonaceous particles, but a consistent increase of +140% of the modulus was achieved when BC400 were added at 20 wt%, probably due to a lower global dimension of these particles, as already shown in previous paragraph. The lower dimension of the particles let us achieve a more homogeneous dispersion, as noticed by SEM analysis, and to increase the interface surface between matrix and BC, thus resulting in a better reenforcing agent.

In Figure 7d, the storage modulus curves, E’, as a function of temperature of all investigated composites are shown. The storage modulus at low temperature, in accordance with static tensile results, increase with increasing the BC load and their pyrolysis temperature, reflecting the same trend of the elastic modulus. Moreover, E’ decrease by increasing the temperature for all samples, but particles presence influences the softening temperature of the samples. At 10% of load, varying the pyrolysis temperature at which the BC particles were obtained does not significantly affect the E’(T) curves, see Figure 7d.

### 3.3. Photo-Oxidation Resistance of Biocomposites

**Figure 8:**
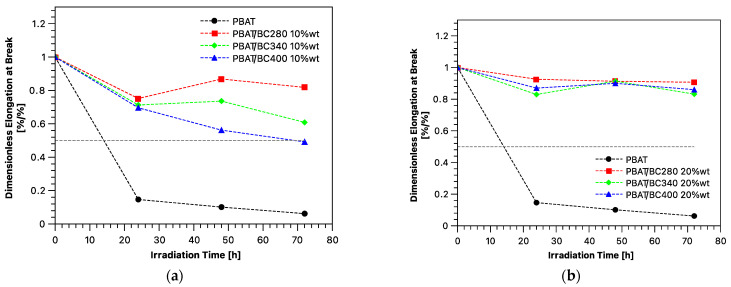

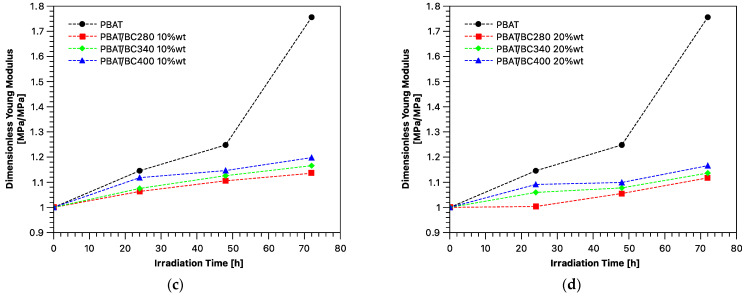
Trends of (**a**,**b**) dimensionless elongation at break and (**c**,**d**) dimensionless Young’s Modulus of PBAT/BC biocomposites as a function of UVB exposure time.

The photo-oxidation behavior is a key parameter to study the oxidative resistance of polymer bio-composites. Thus, the variations of mechanical properties as functions of irradiation time have been monitored. The dimensionless variations of main mechanical properties are reported in Figure 8 Both dimensionless deformation at break and dimensionless Young’s Modulus have been obtained as a ratio between the values at given irradiation time and the value before photo-oxidation exposure. Moreover, it is possible to identify the half time of elongation at break from the dimensionless elongation at break trends, as time at which the value is half of the initial one (evaluated as the time point at which the elongation at break decreases by 50% with respect to the initial value). This parameter is the maximum time at which the polymer films can be still used; after that, the loss of film ductility occurs.

The dimensionless elongation at break of neat PBAT already highlights a significant reduction of its ductility behavior after 24 h of photo-irradiation, showing a half time of 14 h. To sum up, the dimensionless elongation at break curves after a complete cycle of photo-oxidation for biocomposites almost does not achieve the half time of elongation at break. Thus, adding BC particles delay aging phenomena, probably through both UV absorption (being carbonaceous particles) and scavenging action as proved by DPPH assay, discussed before, and according to the literature [35]. This behavior is more pronounced as the filler amount increases. The elongation at break remains almost constant by adding 20 wt% of BC280, and this could be explained considering that the lower the pyrolysis temperature at which the particles are obtained, the higher their radical scavenging efficiency, also according to DPPH assay. In connection with elongation at break, the elastic modulus increases as a function of irradiation time, and this behavior is more pronounced as the decrease of filler load and as the increase of pyrolysis temperature at which the BC was obtained.

**Figure 9:**
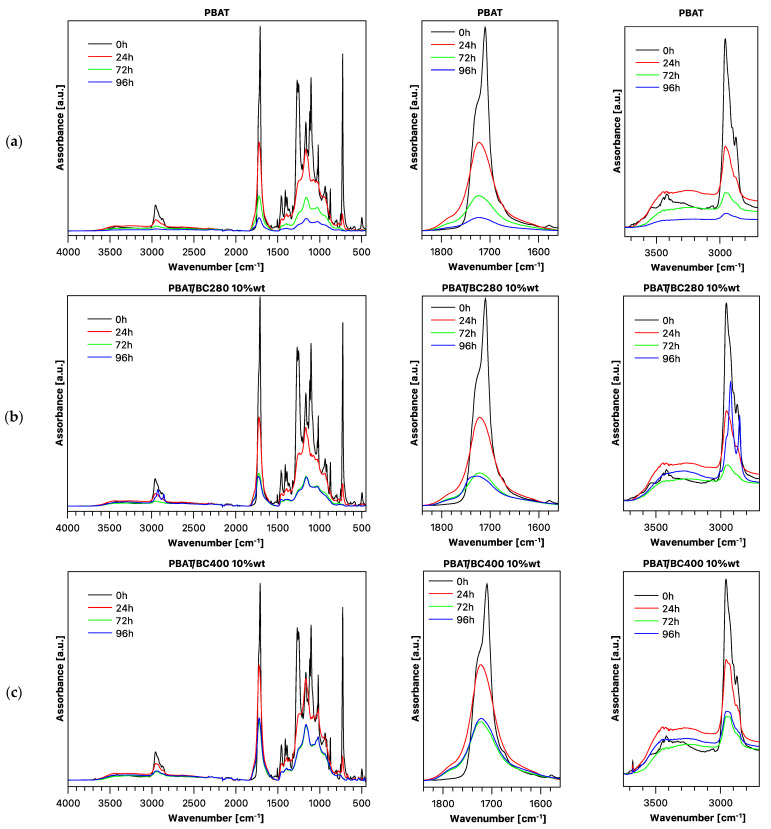
FT-IR spectra in ATR mode as a function of photo-oxidation time of (**a**) neat PBAT, (**b**) PB/BC280 10 wt% and (**c**) PBAT/BC400 10 wt%.

A complementary analysis to monitoring the aging behavior of neat PBAT and PBAT-based composites during weathering test by ATR-FTIR analysis was also carried out. Selected spectra of neat PBAT, PBAT/BC280 10 wt% and PBAT/BC400 10 wt% are shown in Figure 9 with two insets per each spectrum that highlight the main chemical variation occurred during photo-oxidation. The typical bands of PBAT can be recognized in Figure 9a: -OH broad band between 2700 and 3000 cm^−1^, the two peaks related to CH_2_ stretching vibration at 2956 and 2865 cm^−1^, the strong peak of C=O at 1710 cm^−1^, a clear peak representing CH_2_ groups at 720 cm^−1^, and two peaks related to C-O bond in the ester linkage are present at 1275 and 1250 cm^−1^. All these signals tend to change their intensity as a function of UV-irradiation time, highlighting that photodegradation of PBAT matrix occurs.

A reduction of C=O functional groups is clearly visible, since the decreasing and shifting of the neat peak in 1710 cm^−1^, with a creation of a left shoulder, displays the formation of free C=O due to the occurring of chain scission by means of Norrish I scission reaction, according to the literature [35,45]. Moreover, the occurrence of Norrish II reaction can be related to the formation of free OOH and/or peroxide, which lead to the appearance of peaks/sholders at 3410 and 3440 cm^−1^ due to autocatalytic photo-oxidation reactions. For the two composites shown in comparison with neat matrix, chosen for shortness to correlate the two different impacts in variation of mechanical properties, the variation of functional group peaks exposed for neat PBAT matrix appears less intense, giving explanation for better mechanical stability too.

## 4. Conclusions

In this work, the use of a biochar particles, as a pyrolysis product of organic biowaste, was investigated. The biochar particles, as the results of three different pyrolysis process temperatures were added to PBAT matrix, and the properties of produced biocomposites were investigated. The BC carbon content increases as the pyrolysis process temperature increases. At lower pyrolysis temperature, BC particles showed a higher scavenging efficiency, and this is noticeable both by radical scavenging assay for biochar particles and composite degradation studies. In fact, BC280 showed the faster kinetics of scavenging free radicals in DPPH solution, and a better scavenger role during photo-oxidation study. Moreover, the three different size distribution curves are a direct result of the differences in chemical structure and composition related to pyrolysis temperature condition and the consequent fragility, since the milling conditions were the same for the three particles. In particular, the particle dimension decreases as the pyrolysis process temperature increases, and this is noticeable by the composite mechanical properties and rheologic behavior, in which PBAT/BC400 was shown to be the better filler reinforcing agent. Furthermore, BC particles showed a great compatibility and dispersion grade within the PBAT matrix.

## Figures and Tables

**Table 1:** Elemental composition of carob waste and biochar particles.

	%C	%H	%N
Carob Waste	46.94 ± 0.7 ^a^	1.63 ± 0.04 ^a^	5.44 ± 0.04 ^a^
BC 280	65.20 ± 0.2 ^b^	1.75 ± 0.07 ^b^	4.29 ± 0.02 ^b^
BC 340	68.90 ± 0.3 ^c^	1.87 ± 0.09 ^c^	3.79 ± 0.03 ^c^
BC 400	73.10 ± 0.2 ^d^	2.09 ± 0.02 ^d^	3.34 ± 0.06 ^d^

Different letters in the same column indicate significant differences (*p* < 0.05) when analyzed by multiple Student’s *t*-test.

**Table 2:** DSC data from second heating scan.

	Tm [°C]	Tonset [°C]	Toffset [°C]	ΔH [J/g]	χ [%]
PBAT neat	126.7 ± 1.48 ^a^	110.4 ± 1.10 ^a^	141.1 ± 1.2 ^a^	35.5 ± 0.26 ^a^	31.4 ± 0.28 ^a^
PBAT BC280 10wt%	125.5 ± 1.26 ^a^	112.4 ± 2.02 ^a^	144.1 ± 2.39 ^b^	32.8 ± 0.49 ^b^	32.0 ± 0.58 ^a^
PBAT BC340 10wt%	126.1 ± 2.27 ^a^	114.1 ± 1.14 ^a^	138.2 ± 1.28 ^a^	29.8 ± 0.29 ^c^	29.1 ± 0.26 ^b^
PBAT BC400 10wt%	129.5 ± 1.30 ^b^	116.5 ± 1.46 ^b^	141.2 ± 1.57 ^a^	28.2 ± 0.30 ^d^	27.5 ± 0.19 ^c^
PBAT BC280 20wt%	128.3 ± 1.60 ^b^	116.0 ± 1.16 ^b^	142.9 ± 1.33 ^a^	26.1 ± 0.23 ^e^	28.6 ± 0.51 ^d^
PBAT BC340 20wt%	128.3 ± 1.28 ^b^	106.1 ± 1.06 ^c^	139.0 ± 1.09 ^a^	24.1 ± 0.43 ^f^	26.4 ± 0.21 ^e^
PBAT BC400 20wt%	128.7 ± 1.30 ^b^	115.1 ± 1.44 ^b^	142.6 ± 1.35 ^a^	20.1 ± 0.24 ^g^	22.9 ± 0.29 ^f^

Different letters in the same column indicate significant differences (*p* < 0.05) when analyzed by multiple Student’s *t*-tests.

## Data Availability

The data that support the findings of this study are available from the corresponding author upon reasonable request.
